# Lifetime Smoking History and Cause-Specific Mortality in a Cohort Study with 43 Years of Follow-Up

**DOI:** 10.1371/journal.pone.0153310

**Published:** 2016-04-07

**Authors:** Niloofar Taghizadeh, Judith M. Vonk, H. Marike Boezen

**Affiliations:** 1 Department of Epidemiology, University of Groningen, University Medical Center Groningen, Groningen, the Netherlands; 2 Cumming School of Medicine, Division of Respiratory Medicine, University of Calgary, Calgary, Alberta, Canada; 3 GRIAC Research Institute, University of Groningen, University Medical Centre Groningen, Groningen, the Netherlands; University Heart Center Freiburg, GERMANY

## Abstract

**Background:**

In general, smoking increases the risk of mortality. However, it is less clear how the relative risk varies by cause of death. The exact impact of changes in smoking habits throughout life on different mortality risks is less studied.

**Methods:**

We studied the impact of baseline and lifetime smoking habits, and duration of smoking on the risk of all-cause mortality, mortality of cardiovascular diseases (CVD), chronic obstructive pulmonary disease (COPD), any cancer and of the four most common types of cancer (lung, colorectal, prostate, and breast cancer) in a cohort study (Vlagtwedde-Vlaardingen 1965–1990, with a follow-up on mortality status until 2009, n = 8,645). We used Cox regression models adjusted for age, BMI, sex, and place of residence. Since previous studies suggested a potential effect modification of sex, we additionally stratified by sex and tested for interactions. In addition, to determine which cause of death carried the highest risk we performed competing-risk analyses on mortality due to CVD, cancer, COPD and other causes.

**Results:**

Current smoking (light, moderate, and heavy cigarette smoking) and lifetime persistent smoking were associated with an increased risk of all-cause, CVD, COPD, any cancer, and lung cancer mortality. Higher numbers of pack years at baseline were associated with an increased risk of all-cause, CVD, COPD, any cancer, lung, colorectal, and prostate cancer mortality. Males who were lifetime persistent pipe/cigar smokers had a higher risk of lung cancer [HR (95% CI) = 7.72 (1.72–34.75)] as well as all-cause and any cancer mortality. A longer duration of smoking was associated with a higher risk of COPD, any and lung cancer [HR (95% CI) = 1.06 (1.00–1.12), 1.03 (1.00–1.06) and 1.10 (1.03–1.17) respectively], but not with other mortality causes. The competing risk analyses showed that ex- and current smokers had a higher risk of cancer, CVD, and COPD mortality compared to all other mortality causes. In addition, heavy smokers had a higher risk for COPD mortality compared to cancer, and CVD mortality.

**Conclusion:**

Our study indicates that lifetime numbers of cigarettes smoked and the duration of smoking have different impacts for different causes of mortality. Moreover, our findings emphasize the importance of smoking-related competing risks when studying the smoking-related cancer mortality in a general population and that smoking cessation immediately effectively reduces the risk of all-cause and any cancer mortality.

## Introduction

Tobacco smoking has been clearly linked to the most common causes of death including cancer, cardiovascular disease (CVD), and COPD [1–3]. The association between cigarette smoking and the development of these diseases has mainly been explained by the fact that smoking causes a systemic oxidant-antioxidant imbalance and an inflammatory response [4].

Since 1950, many studies have shown that tobacco smoking is associated with an increased risk of lung cancer [5]. In the first International Agency for Research on Cancer (IARC) Monograph on tobacco smoking in 1986, not only lung cancer but also some other types of cancer including cancer of upper aerodigestive tract, pancreas, and lower urinary tract were reported to be associated with smoking [6]. Further studies on the association between smoking and different types of cancers added more cancer types to this list. In the second IARC Monograph on tobacco smoking of 2004 [7] and the last monograph of 2012 [8], 14 other types of cancer were listed as smoking-related cancers, e.g. cancers of the oral cavity, nasal cavity, stomach, liver, kidney, cervix, and myeloid leukemia. However, for some other types of cancer such as prostate and breast cancer still no conclusion on their association with smoking could be drawn across the studies.

Most importantly, like for cancer, tobacco smoking is reported to cause nearly 10% of CVD worldwide [9]. In addition, it has been estimated that 90% of all COPD mortality can be attributed to cigarette smoking [10]. Several prospective cohort studies have addressed the beneficial effect of smoking cessation on CVD and COPD mortality [9,10].

Several studies have indicated that the association between smoking and mortality appears to be different for different causes of death such as cancer, CVD, and COPD [2,11]. However, the differences in this association with smoking between various causes of death (e.g. cancer and CVD) have not been formally analyzed using statistical approaches such as competing risk analyses. So far, the only previous study that statistically analyzed these differences was performed solely on females [11]. Moreover, the majority of previous studies were based on smoking habits at baseline and the level of detail on lifetime smoking history (e.g. regarding duration of smoking and cessation) in these studies is limited. The disadvantage of relying on baseline smoking habits is that the changes in smoking behavior during follow-up cannot be tracked. One example of this behavior change is that baseline smoker may quit during follow-up, therefore this group potentially includes progressively more quitters. Moreover, the results of few studies on lifetime smoking history remain inconclusive. For instance, Vineis et al [12] in a review reported that the beneficial effect of smoking cessation is the same for smoking-related cancer and non-neoplastic disease, while Kenfield et al [11] suggested that benefits of smoking cessation vary by causes of death. Thus, more prospective investigations are needed to determine the lifetime smoking-attributable risk of cause-specific mortality.

In addition, while the majority of studies indicated that females may be more susceptible to the carcinogenic effects of smoking [12–14], little is known about the mechanisms explaining a possible gender difference in the association between smoking and mortality.

Although smoking cigarettes is the most common form of tobacco usage, pipe/cigar smoking also accounts for a considerable proportion of tobacco smokers in some countries. For instance, in the European Prospective Investigation into Cancer (EPIC) study of 500,000 subjects across 10 countries, more than 3,500 males were exclusively pipe/cigar smokers, and 15,000 males smoked pipe/cigar in addition to cigarettes [15]. The EPIC study confirmed that pipe and cigar smoking are related to some cancer types such as lung, bladder, and colon cancer. So far, the relation of pipe/cigar smoking with cause-specific mortality is not as clear as that of cigarette smoking with cause-specific mortality. For instance, Tverdal et al [16] found no or only minor differences in the cause-specific mortality risk between pipe smokers and cigarette smokers, and reported that both are associated with an increased risk of mortality. Another study showed that pipe/cigar smokers have a lower risk for mortality compared with cigarette smokers [17].

We therefore aimed to examine the impact of baseline and lifetime smoking habits, lifetime duration of smoking and duration of smoking cessation on risk of mortality due to all-cause, CVD, COPD and any cancer and the four most common types of cancer, i.e. lung, colorectal, prostate, and breast cancer. We used the Vlagtwedde-Vlaardingen cohort study [18,19], a population-based cohort with follow-up for 43 years which offered the unique possibility to investigate the impact of lifetime smoking habits on the risk of mortality. To shed more light on the role of sex, we additionally assessed whether the association between smoking habits and mortality risk is different for males and females. Other objectives of this study were to assess the association of pipe/cigar smoking for mortality risk, and to determine which cause of death carried the highest risk by performing a competing-risk analysis on mortality due to CVD, COPD, cancer, and other causes.

## Methods

### Ethics statement

The Committee on Human Subjects in Research of the University Medical Center Groningen and University of Groningen reviewed this study and affirmed the safety of the protocol and study design. All participants gave their written informed consent.

### Study population

We investigated the association between lifetime smoking habits and risk of mortality using the Vlagtwedde-Vlaardingen cohort study. The Vlagtwedde-Vlaardingen study is a general population based study on the epidemiology of pulmonary diseases in a general population of exclusively white individuals of Dutch descent [18,19]. The study started in 1965, and participants had medical examinations every 3 years until the final survey in 1989/1990. In Vlaardingen, only participants who were included at baseline (1965 or 1969) were approached for follow-up, whereas in Vlagtwedde new subjects aged between 20 and 65 years were invited to participate at every survey. The number of surveys per subject ranged from one to eight (median number of surveys per subject: five). We updated the vital status of all participants in the Vlagtwedde-Vlaardingen study on December 31st, 2008, and evaluated the following mortality outcomes: all-cause, CVD, COPD, any cancer, and four common types of cancer i.e. lung cancer, colorectal cancer, prostate cancer and breast cancer, either as primary or secondary cause of death. Causes of death were coded according to the International Classification of Diseases (ICD) (see S1 Table for the used ICD-codes) and obtained from Statistics Netherlands (The Hague).

### Population characteristics

At all surveys, we collected data on age, sex, and smoking habits using the Dutch version of the British Medical Research Council questionnaire [18,19]. The BMI (body mass index) was calculated as weight in kilograms divided by the square of the height in meters (kg/m2).

### Smoking habits at baseline

We categorized the smoking habits at baseline (i.e. the first available survey of a subject) into three different categories:

1. Never smokers: subjects who reported never to have smoked any product (cigarettes, pipes or cigars) during their entire life;
2. Ex-smokers: subjects who smoked one or more products but had stopped smoking at least one month before the baseline survey;
3. Current smokers: subjects who smoked at least one cigarette a day or any other product. Current smokers were divided into light (<10 cig/day), moderate (10–20 cig/day) and heavy cigarette smokers (>20 cig/day) and pipe/cigar smokers (only among males). Subjects who smoked both cigarettes and pipe/cigars were classified as cigarette smoker.

Pack-years at baseline were defined as the number of packs of cigarettes (1 pack = 20 cigarettes) smoked per day in one year.

### Lifetime smoking habits

Lifetime smoking habits were determined if subjects participated in at least three surveys. We defined lifetime smoking habits within the interval between the baseline survey and the last available survey as follows:

1. Never smokers: subjects who reported no smoking history at baseline and remained non-smokers during the interval;
2. Ex-smokers: subjects who were ex-smokers at baseline and remained ex-smokers during the interval;
3. Quitters: subjects who were current smokers (cigarette or pipe/cigar) at baseline but successfully quitted smoking and remained quitters during the interval;
4. Persistent smokers: subjects who were current smokers (cigarette or pipe/cigar) at baseline and remained smokers during the interval. Persistent male smokers were further divided into exclusively cigarette smokers, exclusively pipe/cigar smokers, and mixed product smokers (i.e. subjects who smoked pipe/cigar one survey and cigarettes another survey);
5. Unstructured smokers which consists of the remaining subjects.

### Duration of smoking and duration of smoking cessation

We calculated the duration of smoking as the difference between the age at starting smoking and the age at last survey (for persistent smokers) or the age at quitting smoking (for ex-smokers and quitters). We calculated the duration of smoking cessation as the difference between the age at quitting smoking and the age at last survey.

### Statistical analyses

Descriptive analyses of the subject characteristics and the mortality statistics were performed. Independent sample t-tests, Mann-Whitney U tests, or Chi-square tests were used to determine significant differences between groups for continuous and categorical variables, respectively. Multivariate Cox regression with adjustment for age, BMI (at baseline for the analyses on baseline smoking habits and at last survey for all other analyses), sex, and place of residence were used to estimate the hazard ratio of the different smoking habits on the mortality outcomes. Since data on pipe/cigar smoking was available only in males, in the analyses on all subjects we excluded pipe/cigar smokers. To determine whether the association between smoking habits and mortality was different for males and females, stratified analyses were performed and interactions between smoking and sex were tested. To investigate the difference between the never smokers and all other smoking groups we used the never smokers as our reference. In addition, in the analysis on baseline smoking habits we also performed an analysis with the ex-smokers as the reference group in order to investigate the differences between the ex-smokers and the current smokers. In the analysis on lifetime smoking habits we also performed an analysis with the persistent smokers as a reference in order to investigate the differences between the persistent smokers and the ex-smokers and quitters. Two separate analyses on pipe/cigar smoking at baseline and during lifetime were performed in males only. In these analyses pipe/cigar smoking was taken as the reference group. In the analyses on lifetime smoking, both duration of smoking and duration of smoking cessation were entered in one model. To determine the time period in which the hazard ratio after smoking cessation declines, we performed an exploratory analysis on all-cause mortality. We divided the duration of smoking cessation in 5 categories: 1) 0 (reference group: subjects who did not quit smoking), 2) > 0–5, 3) 5–10, 4) 10–15, and > 6) 15 years). In the Cox regression analyses censoring took place when the subjects were still alive, were lost to follow-up, or died from a cause other than the one of interest. Cause-specific mortality was defined on both primary and secondary cause of death and the external causes of death (i.e. suicides, homicides, traffic accidents etc.) were excluded from the analyses, (ICD-9: codes ≥800 and in ICD-10: codes ≥S00). For the analyses on baseline smoking habits, time was defined as years from baseline until mortality or until censoring. For the analyses on lifetime smoking habits, time was defined as years from last survey until mortality or until censoring. Finally, we performed a competing-risk analysis on CVD, COPD, cancer, and other mortality [20]. All analyses were performed at Statistics Netherlands (The Hague, the Netherlands). P-values <0.05 (two-sided) were considered to be statistically significant.

## Results

### Characteristics

Characteristics of the subjects according to vital status on December 31st 2008 are presented in Table 1. Among all 8,465 subjects, 4,505 (53.2%) were alive, 3,675 (43.4%) died due to all-causes mortality (excluding external causes), 150 subjects (1.8%) died due to external causes such as an accident, suicide or homicide. In 13 (0.1%) subjects the cause of death could not be determined, and 122 (1.5%) subjects were lost to follow-up. Of those subjects who died, 1,882 (51.3%) died due to CVD, 1,194 (32.6%) died due to cancer, (147 subjects were coded for both cancer and CVD), 366 (4.3%) died due to COPD (186 subjects were coded for both CVD and COPD, and 83 subjects were coded for both cancer and COPD), and 380 (8.8%) died due to another reason than CVD, COPD or cancer. Of those subjects who died due to cancer, 275 (23.0%) died due to lung cancer, 134 (11.2%) died due to colorectal cancer, 83 (7.0%) died due to prostate cancer, and 117 (9.9%) died due to breast cancer.

**Table 1:** Characteristics at baseline in the general population of Vlagtwedde-Vlaardingen, stratified by vital status after 40 years of follow-up (n = 8465).

Characteristics	Alive (n = 4505) (A)				Mortality			Lost to follow-up (n = 122)	P-value, C vs Alive ^d^	P-value, C vs CVD ^d^	P-value, C vs COPD	P-value, C vs O	P-value, C vs E ^d^
		All-causes (excluding external causes) (n = 3675) (A)	CVD (n = 1882)	COPD (n = 366)	Any cancer (n = 1194) (C)	Another reason than CVD, COPD, or cancer (n = 618) (O)	External causes (n = 150) (E) ^c^						
All subjects, (%) ^a^	53.2	43.4	22.2	4.3	14.1	7.3	1.8	1.4					
Men, n (%)	2121 (47.1)	2053 (55.9)	1071 (56.9)	281 (76.8)	696 (58.3)	218 (45.0)	98 (65.3)	70 (57.4)	0.00	0.22	0.00	0.00	0.06
Age at first survey in years, mean (sd)	30.2 (10.2)	48.8 (10.3)	50.2 (9.6)	50.7 (9.0)	45.9 (11.1)	49.7 (9.8)	43.0 (13.8)	33.2 (13.3)	0.00	0.00	0.00	0.00	0.01
BMI at first survey, kg/m^2^, mean (sd)	24.4 (3.6)	26.7 (4.0)	27.0 (4.1)	25.9 (4.1)	26.3 (4.0)	26.9 (3.9)	24.9 (3.7)	24.0 (3.4)	0.00	0.00	0.16	0.02	0.00
Vlagtwedde, n (%)	3004 (66.7)	2485 (67.7)	1274 (67.7)	268 (73.2)	762 (63.8)	425 (68.8)	110 (73.3)	54 (44.3)	0.06	0.31	0.00	0.04	0.02
**Smoking habits at baseline, n (%)**													
Never smokers	1707 (38.9)	1364 (40.9)	684 (40.3)	49 (13.7)	392 (35.6)	317 (56.5)	50 (37.6)	47 (40.5)					
Ex-smokers	492 (11.2)	294 (8.8)	159 (9.4)	33 (9.2)	100 (9.1)	42 (7.5)	19 (14.3)	19 (16.4)					
Current smokers ^b^									0.00	0.00	0.00	0.00	0.53
Light	908 (20.7)	419 (12.6)	220 (13.0)	45 (12.6)	133 (12.1)	69 (12.3)	17 (12.8)	14 (12.1)					
Moderate	772 (17.6)	715 (21.5)	389 (22.9)	97 (27.2)	242 (22.0)	83 (14.8)	28 (21.1)	19 (14.6)					
Heavy	504 (11.5)	540 (16.2)	246 (14.5)	89 (24.9)	234 (21.3)	50 (8.9)	19 (14.3)	17 (14.7)					
Pack years in ever smoker, median (range)	3.6 (0.5–117.2)	17.4 (0.1–262.2)	18.0 (0.1–111.7)	24.0 (0.5–262.2)	16.2 (0.1–102.9)	15.0 (0.12–82.2)	14.8 (0.1–104.9)	0.5 (0.3–62.0)	0.00	0.07	0.00	0.30	0.06

^a^ All subjects: n = 8465, and in 13 subjects the cause of death could not be determined.

^b^ Current smokers: light cigarette smokers < 10 cig/day, moderate cigarette smokers 10–20 cig/day, and heavy cigarette smokers > 20 cig/day.

^c^ Died due to external causes such as an accident, suicide or homicide.

^d^ P-value calculated by Chi- square, t-test, or Mann-Whitney U test.

The mean age at the first survey of the subjects who died due to CVD was 50.2 (standard deviation (SD) = 9.6) years, and 56.9% were males. The mean age at the first survey of the subjects who died due to COPD was 50.7 (SD = 9.0) years, and 76.8% were males. The mean age at the first survey of the subjects who died due to cancer was 45.9 (SD = 11.1) years, and 58.3% were males. Subjects who died due to cancer had significantly higher BMI levels at the first survey compared to subjects who were alive, but had a lower BMI compared to subjects who died due to CVD. Subjects who died due to cancer were more often heavy smokers (21.3%) compared to subjects who were alive or died due to CVD or causes other than cancer (Table 1). Subjects who died due to cancer were less often heavy smokers compared to subjects who died due to COPD.

Among all 8,465 subjects, 7,361 (87.0%) subjects had data available on baseline smoking habits (the smoking status of this number of subjects was known, and not missing) and on all included covariates (7,075 (83.6%) subjects had data available on cigarette smoking and 286 (3.4%) males had data available on pipe/cigar smoking). In total, 4,325 (51.1%) subjects were ever smokers, and had data on pack-years and on all included covariates. For 3,925 (46.4%) subjects, data on lifetime changes in smoking habits and on all included covariates could be obtained (S2–S4 Tables).

### Baseline smoking habits

Table 2 shows the hazard ratios (with 95% confidence intervals (CI)) of smoking habits at baseline for mortality. Ex-smoking and current smoking (light, moderate, and heavy cigarette smoking) subjects had a higher risk of all-cause, COPD, any cancer and lung cancer mortality, compared to never smokers, the hazard ratios being highest for heavy smokers. The same pattern was observed for mortality due to CVD. No significant associations were found between smoking habits at baseline and risk of colorectal cancer, prostate cancer and breast cancer mortality (Table 2). The analysis with the ex-smokers as the reference showed that light smoking is not associated with a higher risk of all-cause or cause specific mortality compared to ex-smokers (S5 Table).

**Table 2:** Hazard ratio (with 95% confidence interval) of smoking habits at baseline for mortality from all-causes, CVD, COPD, any and specific types of cancer, among all 7074 subjects.

Smoking habits at baseline	All-causes HR (95% CI)	CVD HR (95% CI)	COPD HR (95% CI)	Any cancer HR (95% CI)	Lung cancer HR (95% CI)	Colorectal cancer HR (95% CI)	Prostate cancer HR (95% CI) (males only)	Breast cancer HR (95% CI) (females only)
**All subjects**								
Never smokers	1	1	1	1	1	1	1	1
Ex-smokers	**1.17 (1.00–1.37)**	1.23 (0.98–1.54)	**3.92 (2.18–7.02)**	**1.35 (1.04–1.75)**	**4.59 (2.03–10.37)**	1.58 (0.77–3.27)	0.96 (0.37–2.50)	0.55 (0.17–1.77)
Current smokers ^a^								
light	**1.31 (1.15–1.50)**	**1.42 (1.18–1.72)**	**4.90 (2.96–8.10)**	**1.29 (1.03–1.61)**	**7.06 (3.50–14.23)**	1.60 (0.86–2.96)	0.78 (0.27–2.23)	0.90 (0.50–1.62)
moderate	**1.73 (1.52–1.97)**	**1.90 (1.58–2.30)**	**7.32 (4.42–12.11)**	**1.88 (1.51–2.33)**	**13.06 (6.60–25.74)**	1.40 (0.71–2.77)	0.70 (0.29–1.73)	1.48 (0.74–2.96)
Heavy	**2.04 (1.77–2.36)**	**1.96 (1.58–2.41)**	**10.79 (6.40–18.20)**	**2.73 (2.17–3.43)**	**26.55 (13.40–52.63)**	1.62 (0.75–3.50)	1.05 (0.42–2.66)	1.47 (0.46–4.74)
**Sex-stratified analysis**								
**Effect in females** ^b^								
Never smokers	1	1	1	1	1	1		
Ex-smokers	1.00 (0.74–1.34)	1.16 (0.77–1.73)	**2.02 (0.62–6.64)**	0.86 (0.51–1.45)		1.09 (0.26–4.57)		
Current smokers								
light	**1.20 (1.01–1.43)**	**1.33 (1.04–1.71)**	**4.62 (2.53–8.44)**	1.26 (0.95–1.66)	**7.89 (3.69–16.85)**	0.95 (0.37–2.44)		
moderate	**2.07 (1.67–2.55)**	**1.93 (1.39–2.68)**	**8.23 (4.08–16.61)**	**2.25 (1.63–3.10)**	**9.68 (3.91–23.98)**	**4.26 (1.92–9.45)**		
Heavy	**1.91 (1.34–2.72)**	**2.05 (1.24–3.38)**	**10.69 (4.13–27.67)**	**2.31 (1.37–3.91)**	**25.99 (10.15–66.55)**	3.02 (0.72–12.71)		
**Effect in males** ^c^								
Never smokers	1	1	1	1		1		
Ex-smokers	**1.30 (1.02–1.65)**	1.35 (0.95–1.90)	**7.12 (1.67–30.38)**	**1.68 (1.11–2.54)**	1	1.36 (0.47–3.92)		
Current smokers								
light	**1.50 (1.18–1.91)**	**1.61 (1.14–2.28)**	**7.92 (1.86–33.78)**	1.45 (0.94–2.23)	1.65 (0.76–3.57)	1.85 (0.65–5.24)		
moderate	**1.78 (1.44–2.21)**	**2.05 (1.51–2.79)**	**11.38 (2.79–46.39)**	**1.99 (1.36–2.90)**	**3.88 (2.18–6.92)**	0.74 (0.26–2.13)		
Heavy	**2.18 (1.75–2.72)**	**2.10 (1.53–2.90)**	**17.12 (4.19–69.99)**	**3.06 (2.10–4.46)**	**7.64 (4.33–13.64)**	1.17 (0.41–3.36)		
**Interaction between smoking habits and sex**								
Ex-smoking	1.30 (0.89–1.91)	1.17 (0.68–1.99) 97)	3.52 (0.54–22.99)	**1.95 (1.00–3.80)**	**1**	1.24 (0.21–7.39)		
Current smokers								
Light	1.25 (0.93–1.68)	1.21 (0.79–1.85)	1.71 (0.36–8.23)	1.15 (0.69–1.93)	**0.21 (0.07–0.62)**	1.95 (0.48–8.02)		
Moderate	0.86 (0.64–1.17)	1.06 (0.68–1.66)	1.38 (0.29–6.65)	0.89 (0.54–1.46)	0.40 (0.14–1.18)	**0.17 (0.05–0.66)**		
Heavy	1.15 (0.75–1.74)	1.03 (0.57–1.86)	1.60 (0.29–8.76)	1.32 (0.69–2.53)	**0.29 (0.10–0.88)**	0.39 (0.07–2.30)		
Pack years in ever smoker	**1.01 (1.01–1.01)**	**1.01 (1.00–1.01)**	**1.01 (1.01–1.02)**	**1.01 (1.00–1.02)**	**1.02 (1.01–1.02)**	1.01 (0.99–1.02)	**1.01 (1.00–1.03)**	0.98 (0.92–1.04)
**Sex-stratified analysis**								
**Effect in females**	**1.02 (1.01–1.03)**	**1.02 (1.00–1.03)**	1.03 (1.00–1.06)	**1.03 (1.01–1.05)**	**1.05 (1.02–1.09)**	**1.08 (1.04–1.11)**		
**Effect in males** ^b^	**1.01 (1.01–1.01)**	**1.01 (1.00–1.01)**	1.01 (1.10–1.02)	**1.01 (1.00–1.02)**	**1.02 (1.01–1.02)**	1.00 (0.98–1.02)		
**Interaction between pack years and sex**	0.99 (0.98–1.00)	0.99 (0.97–1.01)	0.98 (0.95–1.01)	**0.98 (0.96–1.00)**	**0.96 (0.94–0.99)**	**0.93 (0.90–0.96)**		

Cox regression with adjustment for age, sex, BMI and place of residence. (Reference category: never smokers. In interaction analyses by sex, for lung cancer in males and females the reference category is ex-smokers+ never smokers).

^a^ Current smokers: light cigarette smokers < 10 cig/day, moderate cigarette smokers 10–20 cig/day, and heavy cigarette smokers > 20 cig/day.

^b^ Females who died due to lung cancer reported no history of being ex-smokers at baseline.

^c^ Males who died due to lung cancer reported no history of being never smokers at baseline.

There was significant interaction between light, and heavy cigarette smoking and sex on risk of mortality due to lung cancer (HR: 0.21 (0.07–0.62), and 0.29 (0.10–0.88) respectively); the effect was more pronounced in females. There was significant interaction between moderate cigarette smoking and sex on risk of mortality due to colorectal cancer (HR: 0.17 (0.05–0.66). No significant interactions were observed between smoking and sex on risk of all-cause, CVD, COPD, or any cancer mortality (Table 2).

The competing risk analyses showed that ex- and current smokers had a higher risk of CVD, COPD, and cancer compared to all other mortality causes. In addition, heavy smokers had a higher risk for cancer mortality compared to CVD or other causes of death, but showed a lower risk for cancer compared to COPD mortality. In all these comparisons, COPD carried the highest risk (S6 Table).

Within ever smokers, a higher number of pack years was associated with an increased risk of all-cause, CVD, COPD, any and lung cancer, and prostate cancer mortality. A higher number of pack years was associated with an increased risk of colorectal cancer mortality only among females (HR: 1.08 (95% CI: 1.04–1.11)). There were no significant associations between pack years and risk of mortality due to breast cancer (Table 2). The numbers of pack years significantly interacted with male sex on risk of mortality due to any cancer (HR: 0.98 (0.96–1.00)), lung cancer (HR: 0.96 (0.94–0.99)), and colorectal cancer (HR: 0.93 (0.90–0.96)).

### Lifetime (changes in) smoking habits

Table 3 shows the hazard ratio (with 95% confidence interval) of lifetime smoking habits for mortality. Persistent cigarette smokers had a higher risk to die of all-causes, CVD, COPD, any cancer, and lung cancer compared to never smokers. Subjects who quitted smoking during follow-up had a higher risk to die of COPD and lung cancer compared to never smokers [(HR: 2.81 (1.12–7.08), and 3.65 (95% CI: 1.32–10.10), respectively]. The analysis with the persistent cigarette smokers as the reference showed that never smokers, persistent ex-smokers, quitters, and unstructured smokers had a lower risk to die of all-cause, CVD, COPD, any cancer, and lung cancer (S7 Table).

**Table 3:** Hazard ratio (with 95% confidence interval) of lifetime smoking habits for mortality from all causes, CVD, COPD, any and specific types of cancer among all 3925 subjects.

Lifetime smoking habits ^a^	All-causes HR (95% CI)	CVD HR (95% CI)	COPD HR (95% CI)	Any cancer HR (95% CI)	Lung cancer HR (95% CI)	Colorectal cancer HR (95% CI)	Prostate cancer HR (95% CI) (males only)	Breast cancer HR (95% CI) (females only)
**All subjects**								
Never smoker	1	1	1	1	1	1	1	1
Persistent ex-smoker	1.02 (0.79–1.33)	1.23 (0.85–1.79)	1.55 (0.43–5.56)	0.98 (0.64–1.49)	1.80 (0.46–6.96)	0.76 (0.22–2.66)	1.04 (0.20–5.37)	1.09 (0.25–4.70)
Quitters	0.99 (0.81–1.21)	1.06 (0.79–1.43)	**2.81 (1.12–7.08)**	1.18 (0.87–1.61)	**3.65 (1.32–10.10)**	0.89 (0.35–2.27)	0.55 (0.11–2.64)	1.49 (0.65–3.42)
Persistent cigarette smokers	**1.92 (1.61–2.30)**	**2.11 (1.61–2.76)**	**8.43(3.56–19.93)**	**2.06 (1.56–2.72)**	**11.45 (4.39–29.87)**	1.22 (0.50–2.99)	0.79 (0.17–3.60)	0.73 (0.28–1.88)
Unstructured	0.92 (0.69–1.23)	1.05 (0.69–1.59)	2.34 (0.67–8.14)	1.05 (0.68–1.62)	1.98 (0.47–8.40)	0.59 (0.13–2.66)	0.91 (0.13–6.55)	0.76 (0.22–2.57)
**Sex-stratified analysis**								
**Effect in females** ^b^								
Never smoker	1	1	NA	1	NA	1		
Persistent ex-smoker	1.16 (0.74–1.84)	1.60 (0.88–2.93)	NA	0.72 (0.29–1.76)	NA	1.20 (0.16–9.27)		
Quitters	0.86 (0.62–1.18)	0.93 (0.58–1.48)	NA	1.06 (0.67–1.69)	NA	0.76 (0.17–3.43)		
Persistent cigarette smokers	**1.85 (1.47–2.34)**	**2.08 (1.48–2.93)**	NA	**1.77 (1.23–2.55)**	NA	1.51 (0.47–4.83)		
Unstructured	0.83 (0.57–1.23)	0.86 (0.48–1.54)	NA	1.04 (0.60–1.80)	NA	0.57 (0.07–4.41)		
**Effect in males** ^c^								
Never smoker	1	1	NA	1	NA	1		
Persistent ex-smoker	1.18 (0.76–1.83)	1.32 (0.68–2.56)	NA	1.63 (0.76–3.49)	NA	0.52 (0.08–3.31)		
Quitters	1.24 (0.83–1.83)	1.27 (0.69–2.33)	NA	1.89 (0.94–3.78)	NA	0.72 (0.15–3.42)		
Persistent cigarette smokers	**2.31 (1.58–3.38)**	**2.44 (1.36–4.39)**	NA	**3.32 (1.69–6.49)**	NA	0.91 (0.20–4.12)		
Unstructured	1.20 (0.72–1.99)	1.44 (0.68–3.05)	NA	1.54 (0.65–3.65)	NA	0.50 (0.05–5.51)		
**Interaction between lifetime smoking habits and sex**								
Never smoker	1	1	NA	1	NA	1		
Persistent ex-smoker	1.02 (0.54–1.92)	0.82 (0.34–2.02)	NA	2.28 (0.70–7.45)	NA	0.43 (0.03–6.98)		
Quitters	1.44 (0.87–2.40)	1.37 (0.64–2.94)	NA	1.78 (0.77–4.10)	NA	0.95 (0.11–8.28)		
Persistent cigarette smokers	1.25 (0.80–1.95)	1.18 (0.59–2.33)	NA	1.87 (0.87–4.05)	NA	0.60 (0.09–4.12)		
Unstructured	1.44 (0.76–2.72)	1.68 (0.65–4.33)	NA	1.48 (0.53–4.13)	NA	0.87 (0.04–20.45)		

Cox regression with adjustment for age (at last visit), sex, BMI (at last visit) and place of residence. (Reference category: never smokers).

^a^ lifetime smoking habits were defined within the interval between the baseline survey and the last available survey as follows: 1) Never smokers: subjects who reported no smoking history at baseline and remained non-smokers during the interval, 2) Ex-smokers: subjects who were ex-smokers at baseline and remained ex-smokers during the interval, 3) Quitters: subjects who were current smokers (cigarette or pipe/cigar) at baseline but successfully quitted smoking during the interval, 4) Persistent smokers: subjects who were current smokers (cigarette or pipe/cigar) at baseline and remained smokers during the interval, and 5) unstructured smokers which consists of the remaining subjects.

^b^ Females who died due to COPD, or lung cancer were not persistent ex-smokers, or unstructured smokers during follow-up.

^c^ Males who died due to cancer were not persistent never smokers during follow-up. NA: The model did not converge.

There were no significant associations between lifetime smoking habits and risk of mortality due to colorectal cancer, prostate cancer, or breast cancer (Table 3). There were no significant interactions between lifetime persistent cigarette smoking and sex on risk of all-cause, or cause specific mortality. The competing risk analyses showed that lifetime persistent smokers had a higher risk of CVD, COPD and cancer compared to other mortality causes (S8 Table). In addition, lifetime persistent smokers had a higher risk of COPD compared to cancer and CVD mortality.

### Pipe/cigar smoking

Table 4 shows the hazard ratios (with 95% confidence intervals) of smoking habits for mortality among males. Males who were never smoker at baseline had a lower risk to die of all-cause, CVD, COPD, and any cancer compared to pipe/cigar smokers. Males who were heavy or moderate smokers had a higher risk to die of all-cause, CVD, COPD, any cancer, and lung cancer compared to pipe/cigar smokers. Lifetime persistent ex-smokers had a lower risk to die of lung cancer compared to pipe/cigar smokers HR: 0.12 (95% CI: 0.03–0.55).

**Table 4:** Hazard ratio (with 95% confidence interval) of smoking habits including pipe/cigar smoking for mortality from all causes, CVD, COPD, any and specific types of cancer, among 3480 males for the analyses on baseline and among 2067 males for the analyses on lifetime smoking habits.

	All-causes HR (95% CI)	CVD HR (95% CI)	COPD HR (95% CI)	Any cancer HR (95% CI)	Lung cancer HR (95% CI)	Colorectal cancer HR (95% CI)	Prostate cancer HR (95% CI)
**Baseline smoking habits**							
Never smokers	**0.71 (0.55–0.91)**	**0.68 (0.48–0.97)**	**0.15 (0.04–0.63)**	**0.63 (0.40–0.97)**	-	0.90 (0.28–2.90)	1.32 (0.46–3.81)
Ex-smokers	0.92 (0.75–1.13)	0.91 (0.69–1.22)	1.11 (0.60–2.04)	1.04 (0.73–1.48)	0.82 (0.39–1.73)	1.37 (0.52–3.60)	1.39 (0.54–3.60)
Current smokers							
light	1.07 (0.87–1.31)	1.10 (0.83–1.47)	1.19 (0.65–2.20)	0.89 (0.61–1.29)	0.80 (0.37–1.74)	1.61 (0.62–4.15)	1.02 (0.35–2.95)
moderate	**1.25 (1.05–1.49)**	**1.39 (1.10–1.77)**	**1.67 (1.02–2.73)**	1.21 (0.89–1.64)	**1.85 (1.03–3.32)**	0.65 (0.25–1.70)	0.93 (0.38–2.32)
Heavy	**1.53 (1.28–1.83)**	**1.40 (1.08–1.80)**	**2.54 (1.54–4.19)**	**1.91 (1.41–2.60)**	**3.77 (2.12–6.70)**	1.01 (0.39–2.65)	1.40 (0.54–3.55)
Pipe/cigar smokers	1	1	1	1	1	1	1
**Lifetime smoking habits**							
Never smoker	0.56 (0.29–1.07)	0.63 (0.22–1.78)	NA	**0.27 (0.11–0.71)**	-	0.48 (0.04–5.45)	0.65 (0.06–7.34)
Persistent ex-smoker	0.66 (0.36–1.19)	0.84 (0.33–2.17)	NA	**0.43 (0.20–0.95)**	**0.12 (0.03–0.55)**	0.42 (0.04–4.16)	0.67 (0.08–5.80)
Quitters	0.68 (0.39–1.20)	0.81 (0.33–2.00)	NA	0.49 (0.24–1.02)	0.35 (0.10–1.18)	0.46 (0.06–3.69)	0.36 (0.04–2.88)
Persistent cigarette smokers	1.32 (0.76–2.30)	1.61 (0.66–3.93)	NA	0.85 (0.42–1.74)	1.04 (0.32–3.33)	0.36 (0.04–2.92)	0.47 (0.06–3.80)
Persistent pipe/cigar smokers	1	1		1	1	1	1
Persistent mixed smokers*	1.17 (0.63–2.15)	1.34 (0.51–3.54)	NA	1.04 (0.47–2.29)	0.71 (0.18–2.77)	0.90 (0.09–8.74)	0.59 (0.05–6.56)
Unstructured	0.66 (0.35–1.27)	0.91 (0.33–2.48)	NA	0.41 (0.17–1.02)	0.31 (0.06–1.55)	0.28 (0.02–4.60)	0.59 (0.05–6.61)

Cox regression with adjustment for age, BMI and place of residence.

* Mixed smokers: subjects who smoked pipe/cigar one survey and cigarettes another survey.

NA: The model did not converge. Males who died due to COPD reported no history of never smokers and of lifetime persistent pipe/cigar smokers.

### Lifetime duration of smoking and duration of smoking cessation

A longer lifetime duration of smoking was associated with a higher risk of mortality from, any cancer and from lung cancer [(any cancer: HR: 1.03 (95% CI: 1.00–1.06), and lung cancer: HR: 1.10 (95% CI: 1.03–1.17)], but not with other causes of death (Table 5). There was a significant interaction between duration of smoking and sex on the risk of mortality due to colorectal cancer (HR: 0.91 (0.83–0.99)); the effect was stronger in females. (Table 5).

**Table 5:** Hazard ratio (with 95% confidence interval) of duration of smoking and of duration of smoking cessation for mortality from all causes, CVD, COPD, and any specific types of cancer.

	All-causes HR (95% CI)	CVD HR (95% CI)	COPD HR (95% CI)	Any cancer HR (95% CI)	Lung cancer HR (95% CI)	Colorectal cancer HR (95% CI)	Prostate cancer HR (95% CI)	Breast cancer HR (95% CI)
**All subjects**								
Duration of smoking	1.01 (0.99–1.03)	1.00 (0.98–1.02)	1.06 (1.00–1.12)	**1.03 (1.00–1.06)**	**1.10 (1.03–1.17)**	0.98 (0.91–1.05)	0.98 (0.89–1.09)	0.99 (0.90–1.09)
Duration of smoking cessation	**0.98 (0.96–0.99)**	**0.97 (0.95–1.00)**	0.97 (0.91–1.04)	0.99 (0.96–1.03)	1.02 (0.95–1.10)	0.96 (0.88–1.04)	0.98 (0.88–1.10)	1.03 (0.92–1.14)
**Sex-stratified analysis**								
**Effect in Females**								
Duration of smoking	1.01 (0.99–1.03)	1.00 (0.98–1.03)	1.05 (0.98–1.12)	1.02 (0.99–1.06)	1.07 (0.99–1.16)	1.04 (0.95–1.14)		
Duration of smoking cessation	**0.97 (0.94–1.00)**	0.98 (0.94–1.01)	0.97 (0.87–1.08)	0.98 (0.94–1.03)	0.84 (0.66–1.08)	1.02 (0.91–1.14)		
**Effect in Males**								
Duration of smoking	1.01 (0.99–1.03)	1.00 (0.98–1.03)	1.06 (1.00–1.12)	**1.03 (1.00–1.06)**	**1.10 (1.03–1.17)**	0.94 (0.87–1.02)		
Duration of smoking cessation	**0.98 (0.96–1.00)**	**0.97 (0.94–1.00)**	0.97 (0.91–1.05)	1.00 (0.96–1.03)	1.03 (0.96–1.11)	0.92 (0.84–1.01)		
**Interaction**								
Duration of smoking	1.00 (0.99–1.02)	1.00 (0.98–1.02)	1.01 (0.96–1.06)	1.01 (0.99–1.04)	1.03 (0.98–1.08)	**0.91 (0.83–0.99)**		
Duration of smoking cessation	1.01 (0.98–1.03)	0.99 (0.96–1.03)	1.01 (0.91–1.11)	1.01 (0.97–1.06)	1.22 (0.96–1.56)	0.91 (0.81–1.02)		

Cox regression with adjustment for age, sex, BMI and place of residence.

## Discussion

In this cohort study we investigated the impact of baseline cigarette and pipe/cigar smoking, pack-years, and lifetime smoking history on the relative risk of all-cause and cause-specific mortality (i.e. CVD, COPD and cancer mortality) in both sexes. Current smoking (light, moderate, and heavy cigarette), and lifetime persistent smoking were associated with an increased risk of all-cause, CVD, COPD, any cancer and lung cancer mortality. A higher number of pack years at baseline was associated with an increased risk of all-cause, CVD, COPD, any cancer, lung cancer and prostate cancer mortality. The effect of smoking on the risk of lung cancer mortality was different for males and females; the effect was more pronounced in females. Moreover, significant interactions between light cigarette smoking and sex, and pack years and sex on risk of mortality due to lung cancer were observed. A longer duration of smoking was associated with an increased risk of mortality due to COPD, any cancer and lung cancer, but not with other causes of death. A longer duration of smoking cessation was associated with a decreased risk of all-cause and CVD mortality. In the competing risk analyses, smoking had the highest association with COPD mortality compared to CVD, cancer, and the other mortality causes. In addition, ex- and current smokers had a higher risk of CVD and cancer compared to all other mortality causes, and heavy smokers had a higher risk for cancer mortality compared to CVD.

Our results are in line with the conclusion of the recent review by IARC [8], and other studies [11,21]. In addition, it helps to expand and clarify the links between smoking and common types of cancer and other causes of mortality in both sexes.

Among the smoking-associated case-specific mortality in our study, COPD had the highest risk. Our results for COPD is in line with the results of a previous study (11), in which authors used competing risks survival analysis to evaluate the associations between smoking and all-cause and cause-specific mortality. Authors reported that the smoking-attributed Hazart Ratios for COPD and lung cancer mortality are much higher than the HRs for total mortality (11).

Lung cancer has been clearly linked to smoking [22,23]. However, there is still a need for more researches to clarify the impact of lifetime smoking habits such as duration of smoking and duration of smoking cessation on lung cancer mortality. We found that longer lifetime duration of cigarette smoking is associated with an increased risk of lung cancer mortality among persistent smokers, ex-smokers and quitters. We found no significant association between duration of smoking cessation and risk of mortality due to lung cancer; however, a negative trend was observed. In contrast, the majority of previous studies showed a clear negative association between smoking cessation and lung cancer. Evidence suggests that this association varies by histologic cell type [24,25]. An increased duration of smoking cessation has been linked to a decreased risk of small cell and squamous cell carcinoma but not with adenocarcinoma [24,25]. Apart from the role of histologic cell type in this association, the time that needs to pass before the risk of lung cancer among quitters reaches that of never smokers is less clear. Bosse et al [25] investigated the effect of smoking cessation on the molecular signature of smoking in lung tissues and showed that smokers who quitted more recently have the same gene-expression profile as current smokers, while over time this profile appears to change into the profile of never smokers. However, the mechanism underlying this association is still unclear and warrants further studies.

One objective of our study was to investigate the potential effect modification of sex on the association between smoking and cause-specific mortality. Our results confirm the hypothesis by Henschke et al [26] that females may be more susceptible to develop lung cancer than males. However, there are also studies showing a higher susceptibility among males [5]. A systematic review and meta-analysis of the epidemiological evidence on the association between lung cancer and smoking showed that the relative risks for smoking tended to be higher for males compared to females. [5]. However, the authors suggested this does not necessarily reflect a higher susceptibility for males, since it could also reflect a higher exposure to occupational carcinogens and a different smoking history (e.g, longer smoking duration) in males [5].

Additionally, we observed that the effect of moderate and heavy smoking compared to ex-smoking on risk of all-cause mortality was more pronounced in females than in males. This may suggest that quitting smoking is more beneficial for females than males in term of lung cancer as well as all-cause mortality. However, a number of questions remain to be answered. For instance, it is still unclear whether the same extent of exposure (number of smoked cigarettes, duration of smoking, and depth of inhalation) carries similar risks in males and females for cause-specific mortality.

Breast cancer mortality was not associated with baseline and lifetime smoking habits, duration of smoking, or duration of smoking cessation. Whether there is an association between smoking and breast cancer remains debated. While the majority of studies reported no association, some studies suggested that this association is limited to pre-menopausal breast cancer and estrogen receptor positive breast cancer [27–33]. Several mechanisms have been proposed for possible protective or carcinogenic effects of cigarette smoking on breast cancer incidence [28–32]. A high level of estrogen is associated with an increased risk of breast carcinogenesis [31], and smoking has an anti-estrogenic capacity. Therefore, one of the proposed mechanisms is that cigarette smoking decreases the risk of breast cancer through this “anti-estrogenic” mechanism. Smoking may enhance the metabolism of estradiol, increase the binding of estrogens by serum sex hormone-binding globulin, and lower the levels of estrogen derived from adipose tissue [32] thus leading to a decreased risk of breast cancer. However, as mentioned earlier, smoking has also been linked to an increased risk of breast cancer [31]. Furthermore, the European Prospective Investigation into Cancer (EPIC) study on the association between smoking and breast cancer suggested that passive smoke exposure also plays a role in this. They reported that the risk estimates of active smoking for breast cancer were higher when the reference group consisted of never smokers who were also not exposed to passive smoke, compared to when passive smoking was not taken into account [33]. There are also suggestions that the association between smoking and breast cancer could be confounded by alcohol. A collaborative reanalysis of individual data from 53 epidemiological studies showed that ever-smokers and current smokers did not have an increased risk of breast cancer compared to never-smokers among women who reported no alcohol consumption [34].

Apart from the above mentioned factors that can affect the link between smoking and cancer, it has been hypothesized that genetic variants in enzymes involved in the metabolism of carcinogens may also play a role in this link. Cigarette smoke contains many carcinogens such as polycyclic aromatic hydrocarbons, nitrosamines, and heterocyclic amines. In particular, the fact that susceptibility to these carcinogens may differ widely among individuals due to genetic variation may explain the observed discrepancy of results among studies. This suggests that the findings from one population for the link between smoking and cancer may not be easily generalizable to another population [33].

Our findings regarding mortality due to CVD are in line with several other studies that reported that smoking is associated with increased risk of mortality due to CVD [1,3]. Interestingly, subjects who quitted smoking during follow-up had no increased risk of mortality due to CVD compared to never smokers. In line with our finding, the majority of previous studies show that after smoking cessation, the risk of CVD mortality declines [35, 36]. This can be explained by the fact that the inflammatory component of CVD resulting from smoking is reversible after smoking cessation [35]. However, the time that needs to pass before the risk of CVD mortality among ex-smokers reaches that of never smokers is less clear. In addition, the level to which the inflammatory response declines after smoking cessation has not been subject of many studies. Therefore more studies are needed on the impact of smoking cessation regarding the risk of CVD mortality as well as other causes of death.

One of our interesting findings regarding the all-cause mortality is that the hazard ratio after smoking cessation declined in the first 5 years after quitting and this effect was not observed any further with a longer duration of smoking cessation. Our in depth analyses showed that at least 3 years is needed to pass before the risk of all-cause mortality declines among ex-smokers (results not shown). However, due to the small sample size our results need to be interpreted with caution.

Our analyses including pipe/cigar smokers among males revealed that heavy cigarette smoking at baseline was associated with an increased risk of all-cause mortality, and mortality due to CVD, any cancer, and lung cancer compared to pipe/cigar smokers. Contrary to the baseline findings, lifetime persistent cigarette smoking was not significantly associated with a higher risk of all-cause or cause-specific mortality compared to pipe/cigar smoking.

Higher concentrations of nitrosamines, which are associated with an increased risk of cancer, are found in pipe/cigar smoke than in cigarette smoke [15–17]. Previous studies [12, 15, 35] suggest that switching to pipe/cigar smoking is not a safe alternative for cigarette smokers to reduce the risk of cancer. Shaper et al [37] investigated the impact of pipe/cigar smoking on all-cause mortality, cancer, and CVD and reported that both pipe/cigar smoking and mixed product smoking was associated with an increased risk of all-cause mortality, cancer incidence, and CVD compared to never smoking. Unfortunately, Shaper et al. did not address lifetime impact of (changes in) pipe/cigar smoking. Given the fact that the types of cigars varies among countries (e.g., different sizes, filters and packaging) more studies are needed to address the detailed information on the types of cigars and the depth of inhalation and to clarify the association between cigar-smoking and cause-specific mortality [38].

One objective of our study was to use competing risk survival analysis to evaluate the impact of baseline and lifetime smoking on causes-specific mortality simultaneously. One of our interesting findings was that heavy smokers had a significantly higher risk to die of cancer than to die of CVD among all subjects, while this pattern was not significant among males.

The major strength of our current study is the longitudinal design. We were able to follow our participants for 43 years, which provided a unique wide time window for evaluating the impact of lifetime (changes in) smoking habits on cause-specific mortality. Another strength of our study is the high follow-up rate, since 98.5% of the included subjects could be traced back [39]. We studied the association between smoking and all-cause, CVD, COPD, and cancer mortality in four common types of cancer (lung cancer, colorectal cancer, prostate cancer and breast cancer), while previous studies investigated overall mortality or any cancer mortality, or only one specific cause of death.

The lack of information on alcohol consumption, inhalation patterns (e.g., depth of inhalation and smoke holding), and menopause status of females may be considered a limitation in our study. Evidence shows that the menopause status of females may modify the effect of smoking on risk of cause-specific mortality [11]. Another drawback of our study is that subjects were not asked about passive exposure to tobacco smoke. Therefore it is possible that some passive smokers were incorrectly classified as unexposed (never smokers). Finally, subjects who smoked both cigarettes and pipe/cigars at baseline were coded as a cigarette smoker in our study. Therefore, the impact of baseline mixed smokers on mortality risk could not be investigated.

In summary, our findings, by addressing the impact of lifetime smoking habits in both sexes, corroborate and expand the ideas of Kenfield et al [11], who, in a study of females, found stronger associations between smoking and mortality when using updated smoking status during follow-up compared to baseline measurements, suggesting that many of the previous studies may have underestimated the risk of mortality due to smoking by considering only single measurements (i.e. at baseline).

In addition, our study suggests that the impact of lifetime cigarette, pipe/cigar smoking and duration of smoking vary for different causes of death, and that smoking cessation is effective to reduce the risk of all-cause mortality.

## Supporting Information

S1 Table ICD-codes for the causes of death. ^a^ Cancer of trachea, bronchus and lung. ^b^ Cancer of colon and rectum (further referred to as colorectal cancer).
(DOC)

S2 Table Number of subjects (%) who died due to all-cause and cause-specific mortality, in the general population of Vlagtwedde-Vlaardingen during 40 years of follow-up according to different smoking habits at baseline. Current smokers: light cigarette smokers < 15 cig/day, moderate cigarette smokers 15–25 cig/day, and heavy cigarette smokers > 15 cig/day.
(DOC)

S3 Table Number of subjects (%) who died due to all-cause and cause-specific mortality, in the general population of Vlagtwedde-Vlaardingen during 40 years of follow-up according to different lifetime smoking habits. Lifetime smoking habits were defined within the interval between the baseline survey and the last available survey as follows: 1) Never smokers: subjects who reported no smoking history at baseline and remained non-smokers during the interval, 2) Ex-smokers: subjects who were ex-smokers at baseline and remained ex-smokers during the interval, 3) Quitters: subjects who were current smokers (cigarette or pipe/cigar) at baseline but successfully quitted smoking and remained quitters during the interval, 4) Persistent smokers: subjects who were current smokers (cigarette or pipe/cigar) at baseline and remained smokers during the interval, and 5) unstructured smokers which consists of the remaining subjects
(DOC)

S4 Table Number of subjects (%) who died due to all-cause and cause-specific mortality, in a general population of Vlagtwedde-Vlaardingen during 40 years of follow-up according to duration of smoking. (DOC)

S5 Table Hazard ratio (with 95% confidence interval) of smoking habits at baseline for mortality from all-causes, CVD, COPD, any and specific types of cancer, among all 7074 subjects (Reference category: ex- smokers). Cox regression with adjustment for age, sex, BMI and place of residence. NA: The model did not converge.
(DOC)

S6 Table Competing risk analysis on the association between smoking habits at baseline and CVD, COPD, cancer, and other mortality. (DOC)

S7 Table Hazard ratio (with 95% confidence interval) of lifetime smoking habits for mortality from all-causes, CVD, COPD, any and specific types of cancer among all 3925 subjects. **(Reference category: persistent smokers).** Cox regression with adjustment for age, sex, BMI and place of residence. NA: The model did not converge.
(DOC)

S8 Table Competing risk analysis on the association between lifetime smoking habits and CVD, COPD, cancer, and other mortality. (DOC)

## Abbreviations

BMI: Body Mass Index
CVD: cardiovascular diseases
HR: Hazard ratio
ICD: International Classification of Diseases
