# Supplementary material for: Lifetime Smoking History and Cause-Specific Mortality in a Cohort Study with 43 Years of Follow-Up
Source: PLoS One. 2016 Apr 7;11(4):e0153310. doi: 10.1371/journal.pone.0153310 (PMC4824471; doi:10.1371/journal.pone.0153310)
Supplement: S1 Table — a Cancer of trachea, bronchus and lung. b Cancer of colon and rectum (further referred to as colorectal cancer). (DOC) [file pone.0153310.s001.doc]

| **ICD-version** | **ICD-7** | **ICD-8** | **ICD-9** | **ICD-10** |
| --- | --- | --- | --- | --- |
|  |  |  |  |  |
| **Years of use** | 1965-1968 | 1969-1978 | 1979-1995 | 1996-2008 |
| **External causes of death** | ≥800 | ≥800 | ≥800 | S, T, V, W, X, Y |
| **Death due to cardiovascular disease** | 330-334, 400-416, 420-422, 430-434, 440-447, 450-456, 460-468, 782.4 | 390-398, 400-404, 410-414, 420-429, 430-438, 440-448 (excl.444.2), 450-458, 782.4 | 390-398, 401-405, 410-417, 420-438, 440-448, 451-459, 785.4 | G45-G46, I00-I15, I20-I28, I30-I52, I60-I69,  I70-I79, I80-I89, I95-I97, I98.2, I98.8, I99, M30-M31, N28.0, R02, R58 |
| **COPD** |  |  | 490-492, 494, 496 | J40-J44, J47 |
| **Death due to cancer** |  |  |  |  |
| **Any cancer** | 140-239 , 294 | 140-239 | 140-239 , 288 | C00-C97, D00-D48 |
| **Lung cancer ^a^** | 162, 163 | 162, 163 | 162, 163, 165 | C33, C34, C38, C39 |
| **Colorectal cancer** ^b^ | 153, 154 | 153, 154 | 153, 154 | C18-C21 |
| **Prostate cancer** | 177 | 185 | 185 | C61 |
| **Breast cancer** | 170 | 174 | 174, 175 | C50 |
