# Supplementary material for: Lifetime Smoking History and Cause-Specific Mortality in a Cohort Study with 43 Years of Follow-Up
Source: PLoS One. 2016 Apr 7;11(4):e0153310. doi: 10.1371/journal.pone.0153310 (PMC4824471; doi:10.1371/journal.pone.0153310)
Supplement: S2 Table — Current smokers: light cigarette smokers < 15 cig/day, moderate cigarette smokers 15–25 cig/day, and heavy cigarette smokers > 15 cig/day. (DOC) [file pone.0153310.s002.doc]

| **Smoking habits at baseline** | **All-causes** | **CVD** | **COPD** | **Any cancer** | **Lung cancer** | **Colorectal cancer** | **Prostate cancer** | **Breast cancer** |
| --- | --- | --- | --- | --- | --- | --- | --- | --- |
|  | **events/censored** | **events/censored** | **events/censored** | **events/censored** | **events/censored** | **events/censored** | **events/censored** | **events/censored** |
|  | n (%)/n (%) | n (%)/n (%) | n (%)/n (%) | n (%)/n (%) | n (%)/n (%) | n (%)/n (%) | n (%)/n (%) | n (%)/n (%) |
| **All subjects** |  |  |  |  |  |  |  |  |
| Never smokers | 994 (37.1)/1688 (62.9) | 498 (18.6)/2184 (81.4) | 34 (1.3)/2648 (98.7) | 307 (11.4)/2375 (88.6) | 12 (0.4)/2670 (99.6) | 43 (1.6)/2639 (98.4) |  |  |
| Ex-smokers | 246 (32.8)/504 (67.2) | 127 (16.9)/623 (83.1) | 24 (3.2)/726 (96.8) | 92 (12.3)/658 (87.7) | 14 (1.9)/736 (98.1) | 13 (1.7)/737 (98.3) |  |  |
| Current smokers ^a^ |  |  |  |  |  |  |  |  |
| Light | 352 (27.7)/917 (72.3) | 175 (13.8)/1094 (86.2) | 38 (3.0)/1232 (97.0) | 121 (9.5)/1148 (90.5) | 28 (2.2)/1241 (97.8) | 17 (1.3)/1252 (98.7) |  |  |
| Moderate | 626 (44.4)/784 (55.6) | 332 (23.5)/1078 (76.5) | 82 (5.8)/1328 (94.2) | 222 (15.7)/1188 (84.3) | 74 (5.2)/1336 (94.8) | 19 (1.3)/1391 (98.7) |  |  |
| Heavy | 447 (46.4)/516 (53.6) | 204 (21.1)/759 (78.8) | 70 (7.3)/893 (92.7) | 200 (20.8)/763 (79.2) | 93 (9.7)/870 (90.3) | 13 (1.3)/950 (98.7) |  |  |
|  |  |  |  |  |  |  |  |  |
| **Females** |  |  |  |  |  |  |  |  |
| Never smokers | 895 (42.3)/1220 (57.7) | 451 (21.3)/1664 (78.7) | 32 (1.5)/2083 (98.5) | 275 (13.0)/1840 (87.0) | 12 (0.6)/2103 (99.4) | 38 (1.8)/2077 (98.2) |  | 73 (3.5)/2042 (96.5) |
| Ex-smokers | 47 (18.3)/210 (81.7) | 25 (9.7)/232 (90.3) | 3 (1.2)/254 (98.8) | 15 (5.8)/242 (94.2) | 0 (0.0)/257 (100.0) | 2 (0.8)/255 (99.2) |  | 3 (1.2)/254 (98.8) |
| Current smokers |  |  |  |  |  |  |  |  |
| Light | 160 (21.2)/596 (78.8) | 78 (10.3)/678 (89.7) | 17 (2.2)/739 (97.8) | 64 (8.5)/692 (91.5) | 16 (2.1)/740 (97.9) | 5 (0.7)/751 (99.3) |  | 15 (2.0)/741 (98.0) |
| Moderate | 101 (28.0)/260 (72.0) | 41 (11.4)/320 (88.6) | 11 (3.0)/350 (97.0) | 45 (12.5)/316 (87.5) | 8 (2.2)/353 (97.8) | 8 (2.2)/353 (97.8) |  | 10 (2.8)/351 (97.2) |
| Heavy | 32 (30.5)/73 (69.5) | 16 (15.2)/89 (84.8) | 5 (4.8)/100 (95.2) | 15 (14.3)/90 (85.7) | 7 (6.7)/98 (93.3) | 2 (1.9)/103 (98.1) |  | 3 (2.9)/102 (97.1) |
|  |  |  |  |  |  |  |  |  |
| **Males** |  |  |  |  |  |  |  |  |
| Never smokers | 99 (17.5)/468 (82.5) | 47 (8.3)/520 (91.7) | 2 (0.4)/565 (99.6) | 32 (5.6)/535 (94.4) | 0 (0.0)/567 (100.0) | 5 (0.9)/562 (99.1) | 7 (1.2)/560 (98.8) |  |
| Ex-smokers | 199 (40.4)/294 (59.6) | 102 (20.7)/391 (79.3) | 21 (4.3)/472 (95.7) | 77 (15.6)/416 (84.4) | 14 (2.8)/479 (97.2) | 11 (2.2)/482 (97.8) | 12 (2.4)/481 (97.6) |  |
| Current smokers |  |  |  |  |  |  |  |  |
| Light | 192 (37.4)/321 (62.6) | 97 (18.9)/416 (81.1) | 21 (4.1)/493 (95.9) | 57 (11.1)/456 (88.9) | 12 (2.3)/501 (97.7) | 12 (2.3)/501 (97.7) | 7 (1.4)/506 (98.6) |  |
| Moderate | 525 (50.0)/524 (50.0) | 291 (27.7)/758 (72.3) | 71 (6.8)/978 (93.2) | 177 (16.9)/872 (83.1) | 66 (6.3)/983 (93.7) | 11 (1.0)/1038 (99.0) | 15 (1.4)/1034 (98.6) |  |
| Heavy | 415 (48.4)/443 (51.6) | 188 (21.8)/670 (78.1) | 65 (7.6)/793 (92.4) | 185 (21.6)/673 (78.4) | 86 (10.0)/772 (90.0) | 11 (1.3)/847 (98.7) | 13 (1.5)/845 (98.5) |  |
| Pipe/cigar smokers | 174 (60.8)/112 (39.2) | 92 (32.2)/194 (67.8) | 21 (7.3)/265 (92.7) | 55 (19.2)/231 (80.8) | 14 (4.9)/272 (95.1) | 7 (2.4)/279 (97.6) | 7 (11.5)/279 (97.6) |  |
|  |  |  |  |  |  |  |  |  |
| **Pack years in ever smoker** |  |  |  |  |  |  |  |  |
| All subjects | 1612 (37.3)/2713 (62.7) | 800 (18.5)/3525 (81.5) | 197 (4.6)/4129 (95.4) | 620 (14.3)/3705 (85.7) | 201 (4.6)/4124 (95.4) | 62 (1.4)/4263 (98.6) |  |  |
| Females | 302 (22.1)/1067 (77.9) | 135 (9.9)/1234 (90.1) | 31 (2.3)/1338 (97.7) | 129 (9.4)/1240 (90.6) | 28 (2.0)/1341 (98.0) | 15 (1.1)/1354 (98.9) |  | 30 (2.2)/1339 (97.8) |
| Males | 1310 (44.3)/1646 (55.7) | 665 (22.5)/2291 (77.5) | 166 (5.6)/2791 (94.4) | 491 (16.6)/2465 (83.4) | 173 (5.9)/2783 (94.1) | 47 (1.6)/2909 (98.4) | 43 (1.5)/2913 (98.5) |  |
|  |  |  |  |  |  |  |  |  |
