# Supplementary material for: Lifetime Smoking History and Cause-Specific Mortality in a Cohort Study with 43 Years of Follow-Up
Source: PLoS One. 2016 Apr 7;11(4):e0153310. doi: 10.1371/journal.pone.0153310 (PMC4824471; doi:10.1371/journal.pone.0153310)
Supplement: S3 Table — Lifetime smoking habits were defined within the interval between the baseline survey and the last available survey as follows: 1) Never smokers: subjects who reported no smoking history at baseline and remained non-smokers during the interval, 2) Ex-smokers: subjects who were ex-smokers at baseline and remained ex-smokers during the interval, 3) Quitters: subjects who were current smokers (cigarette or pipe/cigar) at baseline but successfully quitted smoking and remained quitters during the interval, 4) Persistent smokers: subjects who were current smokers (cigarette or pipe/cigar) at baseline and remained smokers during the interval, and 5) unstructured smokers which consists of the remaining subjects (DOC) [file pone.0153310.s003.doc]

| **Lifetime smoking habit**  ^a^ | **All-causes** | **CVD** | **COPD** | **Any cancer** | **Lung cancer** | **Colorectal cancer** | **Prostate cancer** | **Breast cancer** |
| --- | --- | --- | --- | --- | --- | --- | --- | --- |
|  | **events/censored** | **events/censored** | **events/censored** | **events/censored** | **events/censored** | **events/censored** | **events/censored** | **events/censored** |
|  | n (%)/n (%) | n (%)/n (%) | n (%)/n (%) | n (%)/n (%) | n (%)/n (%) | n (%)/n (%) | n (%)/n (%) | n (%)/n (%) |
| **All subjects** |  |  |  |  |  |  |  |  |
| Never smoker | 252(24.5)/776(75.5) | 112(10.9)/916 (89.1) | 7 (0.7)/1021 (99.3) | 97 (9.4)/931 (90.6) | 5 (0.5)/1023 (99.5) | 14 (1.4)/1014 (98.6) |  |  |
| Persistent ex-smoker | 90 (30.8)/202 (69.2) | 47 (16.1)/245 (83.9) | 4 (1.4)/288 (98.6) | 33 (11.3)/259 (88.7) | 4 (1.4)/288 (98.6) | 4 (1.4)/288 (98.6) |  |  |
| Quitters | 233 (27.5)/613 (72.5) | 108(12.8)/738 (87.2) | 20 (2.4)/826 (97.6) | 104 (12.3)/742 (87.7) | 22 (2.6)/824 (97.4) | 11 (1.3)/835 (98.7) |  |  |
| Persistent smokers | 507 (36.0)/901 (64.0) | 232(16.5)/1176 (83.5) | 65 (4.6)/1343 (95.4) | 214(15.2)/1194 (84.8) | 83 (5.9)/1325 (94.1) | 15 (1.1)/1393 (98.9) |  |  |
| Unstructured | 60 (17.1)/291 (82.9) | 29 (8.3)/322 (91.7) | 4 (1.1)/347 (98.9) | 27 (7.7)/324 (92.3) | 3 (0.9)/348 (99.1) | 2 (0.6)/349 (99.4) |  |  |
|  |  |  |  |  |  |  |  |  |
| **Females** |  |  |  |  |  |  |  |  |
| Never smoker | 223 (27.5)/588 (72.5) | 100 (12.3)/711 (87.7) | 7 (0.9)/804 (99.1) | 88 (10.9)/723 (89.1) | 5 (0.6)/806 (99.4) | 12 (1.5)/799 (98.5) |  | 21 (2.6)/790 (97.4) |
| Persistent ex-smoker | 20 (21.1)/75 (78.9) | 12 (12.6)/83 (87.4) | 2 (2.1)/93 (97.9) | 5 (5.3)/90 (94.7) | 0 (0.0)/95 (100.0) | 1 (1.1)/94 (98.9) |  | 2 (2.1)/93 (97.9) |
| Quitters | 46 (16.1)/240 (83.9) | 22 (7.7)/264 (92.3) | 2 (0.7)/284 (99.3) | 23 (8.0)/263 (92.0) | 1 (0.3)/285 (99.7) | 2 (0.7)/284 (99.3) |  | 8 (2.8)/278 (97.2) |
| Persistent cigarette smokers | 114 (23.9)/363 (76.1) | 53 (11.1)/424 (88.9) | 13 (2.7)/464 (97.3) | 47 (9.9)/430 (90.1) | 17 (3.6)/460 (96.4) | 4 (0.8)/473 (99.2) |  | 6 (1.3)/471 (98.7) |
| Unstructured | 29 (15.3)/160 (84.7) | 13 (6.9)/176 (93.1) | 1 (0.5)/188 (99.5) | 15 (7.9)/174 (92.1) | 0 (0.0)/189 (100.0) | 1 (0.5)/188 (99.5) |  | 3 (1.6)/186 (98.4) |
|  |  |  |  |  |  |  |  |  |
| **Males** |  |  |  |  |  |  |  |  |
| Never smoker | 29 (13.4)/188 (86.6) | 12 (5.5)/205 (94.5) | 0 (0.0)/217 (100.0) | 9 (4.1)/208 (95.9) | 0 (0.0)/217 (100.0) | 2 (0.9)/215 (99.1) | 2 (0.9)/215 (99.1) |  |
| Persistent ex-smoker | 70 (35.5)/127 (64.5) | 35 (17.8)/162 (82.2) | 2 (1.0)/195 (99.0) | 28 (14.2)/169 (85.8) | 4 (2.0)/193 (98.0) | 3 (1.5)194 (98.5) | 6 (3.0)/191 (97.0) |  |
| Quitters | 187 (33.4)/373 (66.6) | 86 (15.4)/474 (84.6) | 18 (3.2)/542 (96.8) | 81 (14.5)/479 (85.5) | 21 (3.8)/539 (96.3) | 9 (1.6)/551 (98.4) | 8 (1.4)/552 (98.6) |  |
| Persistent cigarette smokers | 330 (41.8)/460 (58.2) | 152 (19.2)/638 (80.8) | 40 (5.1)/750 (94.9) | 132 (16.7)/658 (83.3) | 56 (7.1)/734 (92.9) | 7 (0.9)/783 (99.1) | 8 (1.0)/782 (99.0) |  |
| Persistent pipe/cigar smokers | 13 (38.2)/21 (61.8) | 5 (14.7)/29 (85.3) | 0 (0.0)/34 (100.0) | 8 (23.5)/26 (76.5) | 3 (8.8)/31 (91.2) | 1 (2.9)/33 (97.1) | 1 (2.9)/33 (97.1) |  |
| Persistent mixed smokers | 50 (46.7)/57 (53.3) | 22 (20.6)/85 (79.4) | 12 (11.2)/95 (88.8) | 27 (25.2)/80 (74.8) | 7 (6.5)/100 (93.5) | 3 (2.8)/104 (97.2) | 2 (1.9)/105 (98.1) |  |
| Unstructured | 31 (19.1)/131 (80.9) | 16 (9.9)/146 (90.1) | 3 (1.9)/159 (98.1) | 12 (7.4)/150 (92.6) | 3 (1.9)/159 (98.1) | 1 (0.6)/161 (99.4) | 2 (1.2)/160 (98.8) |  |
|  |  |  |  |  |  |  |  |  |

| **Lifetime smoking habit**  ^a^ | **All-causes** | **CVD** | **COPD** |
| --- | --- | --- | --- |
|  | **events/censored** | **events/censored** |  |
|  | n (%)/n (%) | n (%)/n (%) |  |
| **All subjects** |  |  |  |
| Never smoker | 252(24.5)/776(75.5) | 112(10.9)/916 (89.1) |  |
| Persistent ex-smoker | 90 (30.8)/202 (69.2) | 47 (16.1)/245 (83.9) |  |
| Quitters | 233 (27.5)/613 (72.5) | 108(12.8)/738 (87.2) |  |
| Persistent smokers | 507 (36.0)/901 (64.0) | 232(16.5)/1176 (83.5) |  |
| Unstructured | 60 (17.1)/291 (82.9) | 29 (8.3)/322 (91.7) |  |
|  |  |  |  |
| **Females** |  |  |  |
| Never smoker | 223 (27.5)/588 (72.5) | 100 (12.3)/711 (87.7) |  |
| Persistent ex-smoker | 20 (21.1)/75 (78.9) | 12 (12.6)/83 (87.4) |  |
| Quitters | 46 (16.1)/240 (83.9) | 22 (7.7)/264 (92.3) |  |
| Persistent cigarette smokers | 114 (23.9)/363 (76.1) | 53 (11.1)/424 (88.9) |  |
| Unstructured | 29 (15.3)/160 (84.7) | 13 (6.9)/176 (93.1) |  |
|  |  |  |  |
| **Males** |  |  |  |
| Never smoker | 29 (13.4)/188 (86.6) | 12 (5.5)/205 (94.5) |  |
| Persistent ex-smoker | 70 (35.5)/127 (64.5) | 35 (17.8)/162 (82.2) |  |
| Quitters | 187 (33.4)/373 (66.6) | 86 (15.4)/474 (84.6) |  |
| Persistent cigarette smokers | 330 (41.8)/460 (58.2) | 152 (19.2)/638 (80.8) |  |
| Persistent pipe/cigar smokers | 13 (38.2)/21 (61.8) | 5 (14.7)/29 (85.3) |  |
| Persistent mixed smokers | 50 (46.7)/57 (53.3) | 22 (20.6)/85 (79.4) |  |
| Unstructured | 31 (19.1)/131 (80.9) | 16 (9.9)/146 (90.1) |  |
|  |  |  |  |
