# Supplementary material for: Lifetime Smoking History and Cause-Specific Mortality in a Cohort Study with 43 Years of Follow-Up
Source: PLoS One. 2016 Apr 7;11(4):e0153310. doi: 10.1371/journal.pone.0153310 (PMC4824471; doi:10.1371/journal.pone.0153310)
Supplement: S4 Table — (DOC) [file pone.0153310.s004.doc]

|  | **All-causes**  **HR ( 95% CI)** | **CVD**  **HR ( 95% CI)** | **COPD**  **HR ( 95% CI)** | **Any cancer**  **HR ( 95% CI)** | **Lung cancer**  **HR ( 95% CI)** | **Colorectal cancer**  **HR ( 95% CI)** | **Prostate cancer**  **HR ( 95% CI)** | **Breast cancer**  **HR ( 95% CI)** |
| --- | --- | --- | --- | --- | --- | --- | --- | --- |
|  | **events/censored** | **events/censored** | **events/censored** | **events/censored** | **events/censored** | **events/censored** | **events/censored** | **events/censored** |
|  | n (%)/n (%) | n (%)/n (%) | n (%)/n (%) | n (%)/n (%) | n (%)/n (%) | n (%)/n (%) | n (%)/n (%) | n (%)/n (%) |
| **Duration of smoking and cessation** |  |  |  |  |  |  |  |  |
| All subjects | 822 (32.9)/1676 (67.1) | 384 (15.4)/2114 (84.6) | 89 (3.6)/2409 (96.4) | 347 (13.9)/2151 (86.1) | 109 (4.4)/2389 (95.6) | 29 (1.2)/2469 (98.8) |  |  |
| Females | 175 (21.3)/645 (78.7) | 85 (10.4)/735 (89.6) | 17 (2.1)/803 (97.9) | 73 (8.9)/747 (91.1) | 18 (2.2)/802 (97.8) | 7 (0.9)/813 (99.1) |  | 15 (1.8)/805 (98.2) |
| Males | 647 (38.6)/1031 (61.4) | 299 (17.8)/1379 (82.2) | 72 (4.3)/1606 (95.7) | 274 (16.3)/1404 (83.7) | 91 (5.4)/1587 (94.6) | 22 (1.3)/1656 (98.7) | 25 (1.5)/1653 (98.5) |  |

|  | **All-causes**  **HR ( 95% CI)** | **CVD**  **HR ( 95% CI)** | **COPD** |
| --- | --- | --- | --- |
|  | **events/censored** | **events/censored** |  |
|  | n (%)/n (%) | n (%)/n (%) |  |
| **Duration of smoking and cessation** |  |  |  |
| All subjects | 822 (32.9)/1676 (67.1) | 384 (15.4)/2114 (84.6) |  |
| Females | 175 (21.3)/645 (78.7) | 85 (10.4)/735 (89.6) |  |
| Males | 647 (38.6)/1031 (61.4) | 299 (17.8)/1379 (82.2) |  |
