# Supplementary material for: Lifetime Smoking History and Cause-Specific Mortality in a Cohort Study with 43 Years of Follow-Up
Source: PLoS One. 2016 Apr 7;11(4):e0153310. doi: 10.1371/journal.pone.0153310 (PMC4824471; doi:10.1371/journal.pone.0153310)
Supplement: S5 Table — Cox regression with adjustment for age, sex, BMI and place of residence. NA: The model did not converge. (DOC) [file pone.0153310.s005.doc]

| **Smoking habits at baseline** | **All-causes**  **HR ( 95% CI)** | **CVD**  **HR ( 95% CI)** | **COPD**  **HR ( 95% CI)** | **Any cancer**  **HR ( 95% CI)** | **Lung cancer**  **HR ( 95% CI)** | **Colorectal cancer**  **HR ( 95% CI)** | **Prostate cancer**  **HR ( 95% CI)**  **(males only)** | **Breast cancer**  **HR ( 95% CI)**  **(females only)** |
| --- | --- | --- | --- | --- | --- | --- | --- | --- |
| **All subjects** |  |  |  |  |  |  |  |  |
| Never smokers | 0.86 (0.73-1.00) | 0.81 (0.65-1.02) | **0.26 (0.14-0.46)** | **0.74 (0.57-0.96)** | **0.22 (0.10-0.49)** | 0.63 (0.31-1.31) | 0.95 (0.37-2.42) | 1.82 (0.57-5.81) |
| Ex-smokers | 1 | 1 | 1 | 1 | 1 | 1 | 1 | 1 |
| Current smokers ^a^ |  |  |  |  |  |  |  |  |
| light | 1.12 (0.95-1.33) | 1.16 (0.92-1.46) | 1.25 (0.74-2.10) | 0.95 (0.72-1.26) | 1.54 (0.80-2.95) | 1.01 (0.48-2.12) | 0.74 (0.29-1.89) | 1.64 (0.47-5.66) |
| moderate | **1.48 (1.28-1.72)** | **1.55 (1.26-1.90)** | **1.87 (1.18-2.96)** | **1.39 (1.09-1.77)** | **2.84 (1.60-5.04)** | 0.89 (0.44-1.80) | 0.67 (0.31-1.44) | 2.69 (0.74-9.80) |
| Heavy | **1.75 (1.49-2.04)** | **1.59 (1.27-1.99)** | **2.76 (1.72-4.41)** | **2.02 (1.57-2.59)** | **5.78 (3.28-10.19)** | 1.02 (0.47-2.22) | 1.00 (0.45-2.22) | 2.68 (0.54-13.32) |
|  |  |  |  |  |  |  |  |  |
| **Interaction of smoking habits with sex** |  |  |  |  |  |  |  |  |
| **Effect in females** ^b^ |  |  |  |  |  |  |  |  |
| Never smokers | 1.01 (0.75-1.35) | 0.87 (0.58-1.30) | 0.50 (0.15-1.62) | 1.16 (0.69-1.96) | NA | 0.91 (0.22-3.82) |  |  |
| Ex-smokers | 1 | 1 | 1 | 1 | NA | 1 | 1 | 1 |
| Current smokers |  |  |  |  |  |  |  |  |
| light | 1.21 (0.87-1.67) | 1.16 (0.74-1.81) | 2.29 (0.67-7.82) | 1.46 (0.83-2.56) | NA | 0.86 (0.17-4.46) |  |  |
| moderate | **2.08 (1.47-2.94)** | **1.67 (1.02-2.75)** | **4.07 (1.13-14.62)** | **2.61 (1.45-4.68)** | NA | 3.90 (0.83-18.40) |  |  |
| Heavy | **1.92 (1.22-3.01)** | 1.78 (0.95-3.33) | **5.28 (1.26-22.17)** | **2.69 (1.31-5.50)** | NA | 2.76 (0.39-19.66) |  |  |
|  |  |  |  |  |  |  |  |  |
| **Effect in males** ^c^ |  |  |  |  |  |  |  |  |
| Never smokers | **0.77 (0.61-0.98)** | 0.74 (0.53-1.05) | **0.14 (0.03-0.60)** | **0.60 (0.39-0.90)** | NA | 0.74 (0.26-2.12) |  |  |
| Ex-smokers | 1 | 1 | 1 | 1 | NA | 1 | 1 | 1 |
| Current smokers |  |  |  |  |  |  |  |  |
| light | 1.16 (0.95-1.42) | 1.20 (0.91-1.58) | 1.11 (0.61-2.04) | 0.86 (0.61-1.22) | NA | 1.36 (0.60-3.08) |  |  |
| moderate | **1.38 (1.17-1.62)** | **1.52 (1.21-1.91)** | 1.60 (0.98-2.61) | 1.19 (0.91-1.55) | NA | 0.54 (024-1.26) |  |  |
| Heavy | **1.69 (1.42-2.00)** | **1.56 (1.23-1.90)** | **2.41 (1.47-3.95)** | **1.83 (1.40-2.39)** | NA | 0.86 (0.37-1.99) |  |  |
|  |  |  |  |  |  |  |  |  |
| **Interaction** |  |  |  |  |  |  |  |  |
| Never smoker | 0.77 (0.52-1.13) | 0.86 (0.50-1.46) | **0.28 (0.04-1.86)** | **0.51 (0.26-1.00)** | NA | 0.80 (0.14-4.78) |  |  |
| Ex-smoking | 1 | 1 | 1 | 1 | NA | 1 | 1 | 1 |
| Current smokers |  |  |  |  |  |  |  |  |
| Light | 0.96 (0.66-1.41) | 1.04 (0.61-1.76) | 0.49 (0.12-1.92) | 0.59 (0.31-1.14) | NA | 1.57 (0.25-9.83) |  |  |
| Moderate | **0.66 (0.45-0.97)** | 0.91 (0.53-1.57) | **0.39 (0.10-1.55)** | **0.46 (0.24-0.87)** | NA | **0.14 (0.02-0.81)** |  |  |
| Heavy | 0.88 (0.54-1.42) | 0.88 (0.45-1.73) | 0.46 (0.10-2.08) | 0.68 (0.32-1.46) | NA | 0.31 (0.04-2.63) |  |  |
|  |  |  |  |  |  |  |  |  |
