# Supplementary material for: Lifetime Smoking History and Cause-Specific Mortality in a Cohort Study with 43 Years of Follow-Up
Source: PLoS One. 2016 Apr 7;11(4):e0153310. doi: 10.1371/journal.pone.0153310 (PMC4824471; doi:10.1371/journal.pone.0153310)
Supplement: S6 Table — (DOC) [file pone.0153310.s006.doc]

| **Smoking habits at baseline** | **Cancer mortality**  **HR (95% CI)** | **CVD mortality**  **HR (95% CI)** | **COPD mortality**  **HR (95% CI)** | **Other mortality**  **HR (95% CI)** | **Difference**  **CVD vs Cancer**  **HR (95% CI)** | **Difference COPD vs Cancer** | **Difference Other vs Cancer** | **Difference COPD vs CVD** | **Difference Other vs CVD** | **Difference Other vs COPD** |
| --- | --- | --- | --- | --- | --- | --- | --- | --- | --- | --- |
| **All subjects** |  |  |  |  |  |  |  |  |  |  |
| Never smokers | 1 | 1 | 1 | 1 | 1 | 1 | 1 | 1 | 1 | 1 |
| Ex-smokers | **1.42 (1.11-1.81)** | 1.20 (0.98-1.48) | **3.38 (1.99-5.72)** | 0.76 (0.53-1.10) | 0.85 (0.63-1.15) | **2.38 (1.34-4.22)** | **0.54 (0.35-0.83)** | **2.80 (1.60-4.90)** | **0.63 (0.42-0.95)** | **0.23 (0.12-0.43)** |
| Current smokers |  |  |  |  |  |  |  |  |  |  |
| light | **1.47 (1.18-1.83)** | **1.32 (1.10-1.58)** | **4.23 (2.65-6.73)** | 0.99 (0.75-1.33) | 0.90 (0.68-1.18) | **2.87 (1.73-4.78)** | **0.68 (0.47-0.96)** | **3.20 (1.95-5.24)** | 0.75 (0.54-1.05) | **0.24 (0.14-0.41)** |
| Moderate | **1.98 (1.64-2.40)** | **1.83 (1.56-2.15)** | **6.64 (4.42-9.99)** | 0.95 (0.72-1.25) | 0.92 (0.74-1.15) | **3.35 (2.17-5.18)** | **0.48 (0.35-0.66)** | **3.63 (2.38-5.54)** | **0.52 (0.38-0.70)** | **0.14 (0.09-0.23)** |
| Heavy | **2.95 (2.42-3.60)** | **1.86 (1.55-2.24)** | **9.35 (6.14-14.22)** | 0.78 (0.54-1.13) | **0.63 (0.50-0.80)** | **3.17 (2.02-4.95)** | **0.27 (0.18-0.39)** | **5.02 (3.23-7.81)** | **0.42 (0.29-0.62)** | **0.08 (0.05-0.14)** |
|  |  |  |  |  |  |  |  |  |  |  |
| **Females** |  |  |  |  |  |  |  |  |  |  |
| Never smokers | 1 | 1 | 1 | 1 | 1 | 1 | 1 | 1 | 1 | 1 |
| Ex-smokers | 1.07 (0.63-1.80) | 1.09 (0.73-1.63) | 1.86 (0.57-6.06) | 1.05 (0.55-1.98) | 1.02 (0.53-1.97) | 1.74 (0.48-6.33) | 0.98 (0.43-2.23) | 1.70 (0.49-5.94) | 0.96 (0.45-2.04) | 0.56 (0.15-2.17) |
| Current smokers |  |  |  |  |  |  |  |  |  |  |
| light | **1.66 (1.25-2.19)** | 1.21 (0.95-1.55) | **3.57 (1.97-6.46)** | 0.77 (0.50-1.19) | 0.73 (0.51-1.05) | **2.16 (1.12-4.13)** | **0.47 (0.28-0.78)** | **2.95 (1.56-5.59)** | 0.64 (0.39-1.05) | **0.22 (0.10-0.45)** |
| Moderate | **3.14 (2.28-4.33)** | **1.77 (1.28-2.45)** | **6.67 (3.35-13.29)** | 1.23 (0.68-2.22) | **0.56 (0.36-0.88)** | 2.12 (1.00-4.52) | **0.39 (0.20-0.76)** | **3.77 (1.77-8.04)** | 0.70 (0.36-1.35) | **0.19 (0.08-0.45)** |
| Heavy | **2.98 (1.76-5.03)** | **2.01 (1.22-3.33)** | **9.09 (3.53-23.45)** | 0.33 (0.05-2.33) | 0.68 (0.33-1.39) | **3.05 (1.04-8.99)** | **0.11 (0.01-0.84)** | **4.52 (1.55-13.16)** | 0.16 (0.02-1.23) | **0.04 (0.00-0.32)** |
|  |  |  |  |  |  |  |  |  |  |  |
| **Males** |  |  |  |  |  |  |  |  |  |  |
| Never smokers | 1 | 1 | 1 | 1 | 1 | 1 | 1 | 1 | 1 | 1 |
| Ex-smokers | **1.57 (1.04-2.37)** | 1.39 (0.98-1.97) | **6.85 (1.61-29.20)** | 0.65 (0.37-1.13) | 0.89 (0.52-1.52) | 4.37 (0.97-19.75) | **0.41 (0.21-0.82)** | **4.93 (1.11-21.88)** | **0.47 (0.24-0.89)** | **0.09 (0.02-0.45)** |
| Current smokers |  |  |  |  |  |  |  |  |  |  |
| light | 1.38 (0.89-2.12) | **1.60 (1.13-2.26)** | **8.19 (1.92-34.92)** | 1.15 (0.69-1.91) | 1.16 (0.67-2.02) | **5.95 (1.31-27.02)** | 0.84 (0.43-1.63) | **5.13 (1.16-22.81)** | 0.72 (0.39-1.33) | **0.14 (0.03-0.65)** |
| Moderate | **1.86 (1.27-2.70)** | **2.05 (1.51-2.79)** | **11.84 (2.90-48.27)** | 0.84 (0.53-1.34) | 1.11 (0.68-1.80) | **6.39 (1.49-27.35)** | **0.45 (0.25-0.83)** | **5.77 (1.37-24.33)** | **0.41 (0.24-0.72)** | **0.07 (0.02-0.31)** |
| Heavy | **3.04 (2.09-4.42)** | **2.06 (1.50-2.84)** | **16.70 (4.09-68.23)** | 0.76 (0.45-1.26) | 0.68 (0.41-1.11) | **5.50 (1.28-23.61)** | **0.25 (0.13-0.47)** | **8.11 (1.92-34.35)** | **0.37 (0.20-0.67)** | **0.05 (0.01-0.20)** |
|  |  |  |  |  |  |  |  |  |  |  |
| **Packyears** | **1.01 (1.00-1.01)** | **1.01 (1.01-1.01)** | **1.02 (1.01-1.02)** | 1.00 (0.99-1.01) | 1.00 (1.00-1.01) | **1.01 (1.00-1.02)** | 1.00 (0.99-1.01) | **1.01 (1.00-1.01)** | 0.99 (0.99-1.00) | **0.99 (0.98-1.00)** |
| All subjects | **1.03 (1.01-1.05)** | **1.03 (1.01-1.05)** | **1.05 (1.02-1.08)** | 0.98 (0.93-1.03) | 1.00 (0.98-1.03) | 1.02 (0.99-1.06) | 0.95 (0.91-1.01) | 1.02 (0.99-1.06) | 0.95 (0.90-1.00) | **0.93 (0.88-0.99)** |
| Females | 1.01 (1.00-1.01) | **1.01 (1.00-1.01)** | **1.02 (1.01-1.02)** | 1.00 (0.99-1.01) | 1.00 (1.00-1.01) | **1.01 (1.00-1.02)** | 1.00 (0.99-1.01) | **1.01 (1.00-1.01)** | 1.00 (0.99-1.01) | **0.99 (0.98-1.00)** |
