# Supplementary material for: Lifetime Smoking History and Cause-Specific Mortality in a Cohort Study with 43 Years of Follow-Up
Source: PLoS One. 2016 Apr 7;11(4):e0153310. doi: 10.1371/journal.pone.0153310 (PMC4824471; doi:10.1371/journal.pone.0153310)
Supplement: S7 Table — (Reference category: persistent smokers). Cox regression with adjustment for age, sex, BMI and place of residence. NA: The model did not converge. (DOC) [file pone.0153310.s007.doc]

| **Lifetime smoking habits**  ^a^ | **All-causes**  **HR ( 95% CI)** | **CVD**  **HR ( 95% CI)** | **COPD**  **HR ( 95% CI)** | **Any cancer**  **HR ( 95% CI)** | **Lung cancer**  **HR ( 95% CI)** | **Colorectal cancer**  **HR ( 95% CI)** | **Prostate cancer**  **HR ( 95% CI)**  **(males only)** | **Breast cancer**  **HR ( 95% CI)**  **(females only)** |
| --- | --- | --- | --- | --- | --- | --- | --- | --- |
|  |  |  |  |  |  |  |  |  |
| **All subjects** |  |  |  |  |  |  |  |  |
| Never smoker | **0.52 (0.44-0.62)** | **0.47 (0.36-0.62)** | **0.12 (0.05-0.28)** | **0.49 (0.37-0.64)** | **0.09 (0.03-0.23)** | 0.82 (0.34-2.00) | 1.27 (0.28-5.77) | 1.38 (0.53-3.58) |
| Persistent ex-smoker | **0.53 (0.42-0.67)** | **0.59 (0.43-0.81)** | **0.18 (0.07-0.51)** | **0.47 (0.33-0.69)** | **0.16 (0.06-0.43)** | 0.62 (0.19-1.97) | 1.31 (0.46-3.74) | 1.50 (0.30-7.48) |
| Quitters | **0.52 (0.44-0.61)** | **0.50 (0.40-0.63)** | **0.33 (0.20-0.56)** | **0.57 (0.45-0.73)** | **0.32 (0.20-0.51)** | 0.73 (0.33-1.61) | 0.69 (0.27-1.75) | 2.05 (0.70-6.00) |
| Persistent cigarette smokers | 1 | 1 | 1 | 1 | 1 | 1 | 1 | **1** |
| Unstructured | **0.48 (0.37-0.63)** | **0.50 (0.34-0.74)** | **0.28 (0.10-0.77)** | **0.51 (0.34-0.76)** | **0.17 (0.05-0.55)** | 0.48 (0.11-2.16) | 1.15 (0.25-5.23) | 1.04 (0.26-4.21) |
|  |  |  |  |  |  |  |  |  |
| **Interaction of lifetime smoking habits**  **with sex**  **Effect in females** ^b^ | | |  |  | | | | |
| Never smoker | **0.54 (0.43-0.68)** | **0.48 (0.34-0.68)** | NA | **0.57 (0.39-0.81)** | NA | 0.66 (0.21-2.12) |  |  |
| Persistent ex-smoker | 0.63 (0.39-1.01) | 0.77 (0.41-1.45) | NA | 0.40 (0.16-1.02) | NA | 0.79 (0.09-7.15) |  |  |
| Quitters | **0.46 (0.33-0.65)** | **0.45 (0.27-0.74)** | NA | **0.60 (0.36-0.99)** | NA | 0.51 (0.09-2.79) |  |  |
| Persistent cigarette smokers | 1 | 1 | 1 | 1 | 1 | 1 |  | - |
| Unstructured | **0.45 (0.30-0.68)** | **0.41 (0.23-0.76)** | NA | 0.59 (0.33-1.05) | NA | 0.38 (0.04-3.42) |  |  |
|  |  |  |  |  |  |  |  |  |
| **Effect in males** ^c^ |  |  |  |  |  |  |  |  |
| Never smoker | **0.43 (0.30-0.63)** | **0.41 (0.23-0.74)** | **0.13 (0.05-0.33)** | **0.30 (0.15-0.59)** | NA | 1.10 (0.24-5.01) |  |  |
| Persistent ex-smoker | **0.51 (0.40-0.66)** | **0.54 (0.37-0.78)** | **0.49 (0.11-2.17)** | **0.49 (0.33-0.74)** | NA | 0.57 (0.15-2.20) |  |  |
| Quitters | **0.53 (0.45-0.64)** | **0.52 (0.40-0.67)** | **0.15 (0.03-0.67)** | **0.57 (0.44-0.75)** | NA | 0.80 (0.33-1.95) |  |  |
| Persistent cigarette smokers | 1 | 1 | 1 | 1 | 1 | 1 |  |  |
| Unstructured | **0.52 (0.36-0.75)** | **0.59 (0.35-0.98)** | **0.13 (0.02-1.02)** | **0.46 (0.26-0.83)** |  | 0.55 (0.07-4.25) |  |  |
| **Interaction**  **smoking habits with sex** |  | |  |  | | | |  |
| Never smoker | 0.80 (0.51-1.25) | 0.85 (0.43-1.68) | NA | 0.53 (0.25-1.15) | NA  NA | 1.66 (0.24-11.40) |  |  |
| Persistent ex-smoker | 0.81 (0.47-1.40) | 0.70 (0.34-1.45) | NA | 1.22 (0.45-3.33) | NA | 0.72 (0.06-9.34) |  |  |
| Quitters | 1.15 (0.79-1.69) | 1.16 (0.66-2.03) | NA | 0.95 (0.54-1.67) | NA | 1.58 (0.23-10.69) |  |  |
| Persistent cigarette smokers | 1 | 1 | 1 | 1 | 1 | 1 |  |  |
| Unstructured | 1.15 (0.67-1.99) | 1.42 (0.64-3.15) | NA | 0.79 (0.35-1.80) | NA | 1.44 (0.07-29.04) |  |  |
|  |  |  | |  |  |  |  |  |
