# Supplementary material for: Lifetime Smoking History and Cause-Specific Mortality in a Cohort Study with 43 Years of Follow-Up
Source: PLoS One. 2016 Apr 7;11(4):e0153310. doi: 10.1371/journal.pone.0153310 (PMC4824471; doi:10.1371/journal.pone.0153310)
Supplement: S8 Table — (DOC) [file pone.0153310.s008.doc]

| **Lifetime smoking habits ^a^** | **Cancer mortality**  **HR (95% CI)** | **CVD mortality**  **HR (95% CI)** | **COPD mortality** | **Other mortality**  **HR (95% CI)** | **Difference**  **CVD vs Cancer**  **HR (95% CI)** | **Difference COPD vs Cancer** | **Difference Other vs Cancer** | **Difference COPD vs CVD** | **Difference Other vs CVD** | **Difference Other vs COPD** |
| --- | --- | --- | --- | --- | --- | --- | --- | --- | --- | --- |
| **All subjects** |  |  |  |  |  |  |  |  |  |  |
| Never smoker | 1 | 1 | 1 | 1 | 1 | 1 | 1 | 1 | 1 | 1 |
| Ex-smoker | 0.96 (0.64-1.44) | 1.18 (0.83-1.68) | 1.60 (0.47-5.50) | 0.85 (0.48-1.53) | 1.23 (0.73-2.08) | 1.67 (0.46-6.09) | 0.89 (0.44-1.79) | 1.36 (0.38-4.86) | 0.72 (0.37-1.41) | 0.53 (0.14-2.07) |
| Quitters | 1.14 (0.85-1.52) | 1.02 (0.77-1.35) | **3.05 (1.28-7.24)** | **0.62 (0.39-0.99)** | 0.90 (0.61-1.32) | **2.68 (1.08-6.61)** | **0.54 (0.32-0.93)** | **2.98 (1.21-7.34)** | 0.61 (0.36-1.03) | **0.20 (0.08-0.54)** |
| Persistent | **2.10 (1.63-2.72)** | **1.98 (1.55-2.53)** | **8.77 (4.00-19.23)** | 1.21 (0.83-1.77) | 0.94 (0.68-1.31) | **4.17 (1.84-9.42)** | **0.58 (0.37-0.90)** | **4.43 (1.97-9.98)** | **0.61 (0.40-0.95)** | **0.14 (0.06-0.33)** |
| Unstructured | 1.08 (0.70-1.66) | 1.01 (0.67-1.52) | 2.23 (0.65-7.63) | 0.54 (0.24-1.18) | 0.93 (0.52-1.68) | 2.06 (0.56-7.57) | 0.50 (0.20-1.22) | 2.22 (0.61-8.09) | 0.53 (0.22-1.30) | 0.24 (0.06-1.04) |
|  |  |  |  |  |  |  |  |  |  |  |
| **Females** |  |  |  |  |  |  |  |  |  |  |
| Never-smoker | 1 | 1 | 1 | 1 | 1 | 1 | 1 | 1 | 1 | 1 |
| Ex-smoker | 0.76 (0.31-1.87) | 1.56 (0.85-2.84) | 3.75 (0.78-18.09) | 1.28 (0.46-3.59) | 2.06 (0.70-6.07) | 4.95 (0.81-30.35) | 1.69 (0.43-6.65) | 2.41 (0.45-12.96) | 0.82 (0.25-2.71) | 0.34 (0.05-2.24) |
| Quitters | 1.10 (0.70-1.75) | 0.94 (0.59-1.49) | 1.18 (0.24-5.68) | 0.54 (0.21-1.36) | 0.85 (0.44-1.63) | 1.07 (0.21-5.50) | 0.49 (0.17-1.38) | 1.26 (0.24-6.47) | 0.57 (0.20-1.62) | 0.46 (0.07-2.83) |
| Persistent | **2.00 (1.39-2.88)** | **1.96 (1.39-2.77)** | **6.94 (2.75-17.50)** | 1.38 (0.75-2.51) | 0.98 (0.60-1.60) | **3.47 (1.30-9.32)** | 0.69 (0.35-1.38) | **3.54 (1.33-9.43)** | 0.70 (0.36-1.39) | **0.20 (0.07-0.59)** |
| Unstructured | 1.11 (0.64-1.92) | 0.84 (0.47-1.49) | 0.93 (0.11-7.54) | 0.33 (0.08-1.36) | 0.75 (0.34-1.67) | 0.84 (0.10-7.29) | 0.30 (0.06-1.35) | 1.11 (0.13-9.75) | 0.39 (0.09-1.82) | 0.35 (0.03-4.45) |
|  |  |  |  |  |  |  |  |  |  |  |
| **Males** |  |  |  |  |  |  |  |  |  |  |
| Never smoker | No convergence | | | | | | | | | |
| Ex-smoker |
| Quitters |
| Persistent |
| Unstructured |
